# Using sub-limb observations to measure gravity waves excited by convection

**DOI:** 10.1038/s41526-023-00259-2

**Published:** 2023-02-08

**Authors:** Corwin J. Wright, Jörn Ungermann, Peter Preusse, Inna Polichtchouk

**Affiliations:** 1 Centre for Space, Atmospheric and Oceanic Science, University of Bath, Bath, UK; 2 Forschungszentrum Jülich, Jülich, Germany; 3 European Centre for Medium-Range Weather Forecasts, Reading, UK

**Keywords:** Environmental sciences, Optics and photonics

## Abstract

Convective gravity waves are a major driver of atmospheric circulation, including the stratospheric and mesospheric quasi-biennial oscillation (QBO) and the Brewer–Dobson circulation. Previous work shows clear evidence that these waves can be excited by both single convective cells and by mesoscale convective complexes acting as a single unit. However, the partitioning of the generated waves and, crucially for atmospheric model development, the flux of momentum they transport between these two types of excitation process remains highly uncertain due to a fundamental lack of suitable observations at the global scale. Here, we use both theoretical calculations and sampled output from a high-resolution weather model to demonstrate that a satellite instrument using a sub-limb geometry would be well suited to characterising the short-vertical short-horizontal gravity waves these systems produce, and hence to provide the scientific knowledge needed to identify the relative wave-driving contribution of these two types of convective wave excitation.

## Introduction

One of the most important drivers of atmospheric circulation is the integrated effect of small-scale atmospheric gravity waves (GWs). These waves are commonly generated at near-surface altitudes and then propagate upwards, transporting the momentum and energy needed to drive and control dynamical and chemical processes in the upper troposphere, stratosphere and above. They play a diverse range of roles, including acting as a major control on the speed of the jet streams^1,2^, causing clear air turbulence which affects aviation^3^, and, by coupling into the charged ionosphere above ~90 km altitude, disrupting GPS and radio signals^4^.

GWs are generated by many processes, including wind flowing over mountains (‘orographic generation’) and geostrophic adjustment in the atmosphere. One of the most important and hard to study, however, is GW generation by convective weather systems. These systems are by far the largest source of GWs at tropical latitudes, and also one of the major sources at midlatitudes in all seasons except winter (when orographic generation is dominant at middle and high latitudes).

Convection excites GWs via different processes and at different wavelengths. Past work using case studies has in general focused on wave excitation by single convective cells^5,6^ and upscaled their findings from convective source modelling to global distributions^7^. According to these simulations, the major part of the momentum flux transported by convective GWs is expected to be found at vertical wavelengths *λ*_*z*_ ≤ 10 km and *λ*_*h*_ ≪ 100 km. However, convection often becomes organised and forms in the tropics and subtropics mesoscale convective clusters, which act as wave sources as a whole and excite GWs of much longer horizontal wavelengths^8^. Full-complexity very high-resolution global ECMWF IFS simulations^9^ point to the coexistence of both processes. This, however, is ill-constrained by observations.

Characterising this partitioning is important for understanding the dynamic structure of the middle atmosphere. This is because GWs at low latitudes are vital to the driving of three major atmospheric circulations with important implications for understanding and better forecasting both weather and climate: the Brewer–Dobson circulation (BDC), the mesospheric residual circulation (MRC), and the quasi-biennial oscillation (QBO). The BDC is a key middle-atmospheric circulation which transports chemicals and trace gases from equator to pole in the stratosphere and the MRC is a similar circulation from pole to pole in the mesosphere, while the QBO is a pattern of alternating eastward and westward winds in the tropics which repeats on an irregular cycle averaging 28 months in duration and which is believed to be one of the sources of skill for seasonal-timescale weather forecasts providing memory that short-term weather does not have^10^.

Each of these circulations has important impacts on surface weather and climate, and all three are well known to be driven by waves. The exact partitioning of this wave driving between large-scale waves which are well resolved by current-generation models and smaller-scale GWs is however poorly understood, and in particular, the way this division will evolve in the future is highly uncertain. In particular, there are indications that the QBO is affected by climate change. While the QBO was stable from when it was first observed in the 1950s until 2016, since then two major unexpected disruptions have occurred^11,12^. To understand and predict the future of the QBO, a better understanding of its fundamental driving forces is essential. For the BDC and MRC meanwhile, while the winter BDC is primarily driven by planetary-scale waves, the summer BDC^13^ and the MRC^14^ are driven mainly by GWs. As such, uncertainties in the BDC due to the interaction of different processes and in particular due to missing GW drag prevent models from consistently predicting how it will evolve in coming decades^15–18^.

The portion of the GW spectrum visible to a space observation technique is governed by the observation geometry, the optical depth of the considered emission and the field of view of the instrument. The different observational geometries suitable for this task, as described in ref. ^19^, are typically grouped into limb, nadir and sub-limb sounding. Limb observations, which cover a wide range from short to long vertical wavelengths, have facilitated momentum budget studies of GWs from MCCs with wavelengths *λ*_*h*_ ≥ 100 km^20–22^. Nadir observations, meanwhile, are sensitive only to long vertical wavelengths (*λ*_*z*_ > 15 km). This leaves the postulated major portion^7^ of parameterised CGW momentum flux, at scales both short in the horizontal and in the vertical, uncovered. This gap in our observational coverage is a major problem since it leaves us uncertain as to how the momentum flux these GWs transport is partitioned between single-tower and MCC sources.

**Fig. 1:**
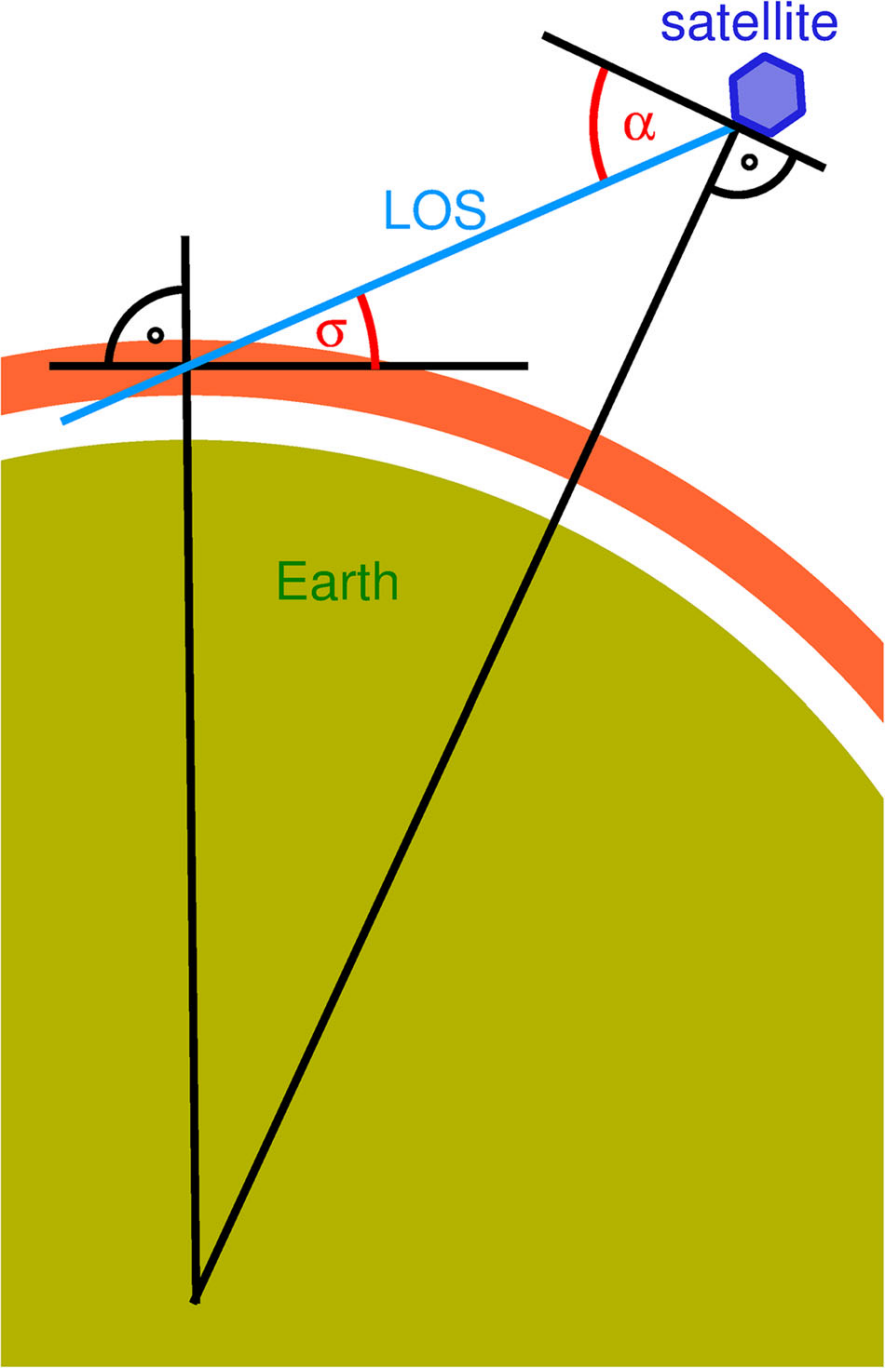
Sketch of viewing geometry. From a position in orbit, the instrument views downward by an angle *α* below the local horizontal. Infrared thermal radiation is emitted from the saturation layer (indicated by the bright-red ring segment above the Earth).

At a physical level, limb sounding requires optically thin emissions and the observable scales are limited by the integration of radiance along the line of sight (LOS). Nadir sounding, meanwhile, is based on radiances saturating around a target altitude, with the vertical resolution limited by the width of this saturation. In the sub-limb, however, while emissions also saturate along the LOS, the sensitivity to GW wavelength is controlled by the alignment of the 3D GW phase fronts with the LOS. This geometry is illustrated in Fig. 1: here, the sub-limb viewing angle *α* and the flight altitude determine the angle *σ* which the LOS forms with the local horizontal. *σ* = 0 corresponds to the highest tangent height and marks the limit between sub-limb and limb; for a flight altitude of 400 km, it is reached for *α* close to 20°.

In the past, this sub-limb geometry was mainly used by microwave instruments (ref. ^23^, and references therein) where the comparably large vertical field of view limited sensitivity. An important exception to this was the astronomical Midcourse Space Experiment (MSX), which observed 4.3 μm CO_2_ emissions and occasionally used a sub-limb mode. Out of 80 such scenes (of 20 minutes’ duration) recorded by MSX, a number contained GW signals and one was analysed in detail^24^ showing signatures of GWs of ~50 km horizontal wavelength arising from a thunderstorm. At the time of the MSX observations, such linear 1D arrays sensitive to the near-infrared were very advanced technology, but since then both detector and cryo-cooler technology have greatly advanced. This allows for the compact and relatively low-priced realisation of even more powerful approaches which would allow the measurement of both a high number of pixels across track as well as a multitude of angles at the same time.

In this paper, we propose an observation concept to study this missing part of the GW spectrum using spaceborne measurements of mid-infrared emissions, based on the saturated emission approach in ref. ^24^. To assess the potential performance of an instrument of this type, we carry out idealised simulations and then sample a high-resolution weather forecasting model with the expected observational characteristics of such an instrument. Finally, we describe the technological underpinnings needed for such a sounder.

## Results

### Observational filter

We first estimate how well the envisioned instrument would capture plain sinusoidal GWs depending on their horizontal and vertical scales. To do so, we initially generate a simplified atmosphere with a climatological midlatitude temperature and CO_2_ profile, working on a horizontal grid of 1 × 1 km spacing with a vertical step of 100 m. This temperature is then perturbed with a sinusoidal monochromatic wave of temperature amplitude 10 K, and the resulting temperature perturbations are retrieved using the JURASSIC2 forward model (see “Methods”). The simulations are then repeated with systematically varying vertical and horizontal wavelengths. Using this perturbed atmosphere, we compute the brightness temperature the satellite would measure at varying sub-limb angles, extracting one brightness temperature estimate per kilometre in the horizontal.

**Fig. 2:**
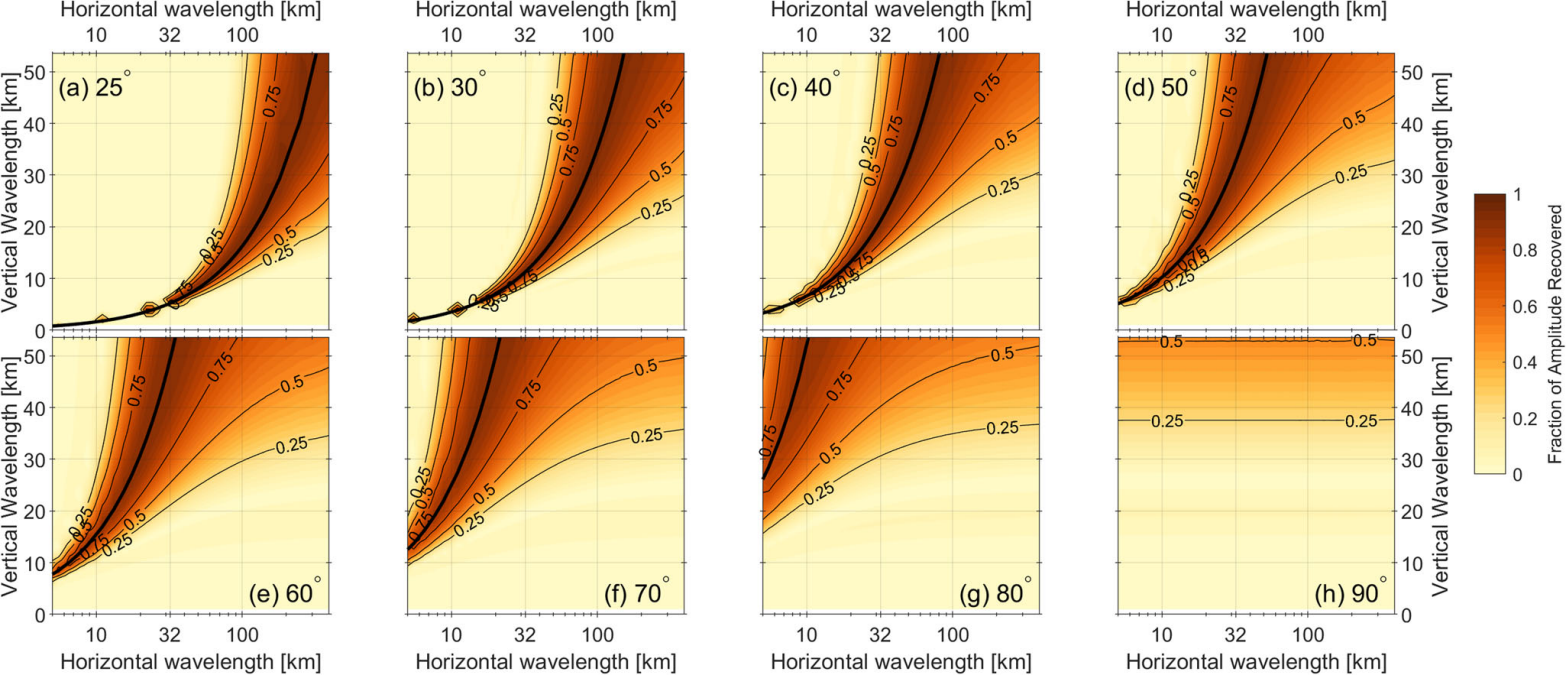
Estimated effect of the observational filter for selected sub-limb viewing angles from 25 to 90°. **a**–**h** Colours and line contours show the fraction of the input signal amplitude recovered for GWs of this horizontal and vertical wavelength. The thick solid line shows the expected optimum visibility where the LOS angle *σ* aligns with the orientation of the phase fronts given by the ratio of vertical and horizontal wavelength.

The simulated brightness temperature measurements are then analysed with a simple least squares fit for the largest sinusoidal frequency, using the results of a Fast Fourier Transform as an initial guess. Finally, the amplitude of this GW signal is computed, and the ratio between the true amplitude of 10 K and the derived value is shown in Fig. 2 across a range of wavelengths at eight selected sub-limb viewing angles. We find no significant bias between the derived wavelength and true horizontal wavelength for all simulations with sufficient sensitivity to extract such estimates.

Convective GWs have typically vertical wavelengths of the order of 6–15 km^13^, corresponding to a phase speed range of 20–50 m/s. From Fig. 2, we see that we are sensitive to these wavelengths at all *σ* between 25 and 60°, but that this sensitivity vanishes for 80° and nadir. We can assess this by considering a fixed vertical wavelength, e.g., 10 km—at such a specified value, the region of maximal sensitivity sweeps the horizontal wavelength range, maximising at ~*λ*_*h*_ = 50 km for 25° and *λ*_*h*_ = 7 km for 60°. Thus, covering the wave field with a wide range of *σ*, one can estimate the aspect ratio of the GWs, and from the horizontal structure determine the horizontal wavelength. In the ideal case of monochromatic waves, this would also allow us to infer the vertical wavelengths by combining the horizontal wavelength and the sensitivity dependence.

We now proceed to consider two more realistic GW cases, both derived from a very high-resolution global numerical weather prediction model simulation.

### Model

We use the output from a TCo7999L137 (~1.4 km horizontal, 137-level vertical) run of the hydrostatic ECMWF Integrated Forecasting System (IFS)^25^, using a 60-s model timestep. This simulation has the highest spatial resolution ever used in a seasonal-timescale global atmospheric model and represents an exceptional scientific resource for studies of this nature. In particular, it has a substantially higher spatial resolution than the global-scale observing system simulation experiment ‘nature runs’ often used in satellite development studies^26^. Because the IFS simulation used only resolves hydrostatic waves, our analysis only considers these waves.

Compared to lower resolution versions of the IFS, this model resolves a much larger part of the GW spectrum, with equal contribution of small-scale GWs with *λ*_*h*_ < 100 km versus larger scale GWs with *λ*_*h*_ > 100 km to the convectively generated GW momentum flux^9^. The model runs without GW or convective parameterisations, and thus all GWs in the model are explicitly generated and propagate freely on the model grid. The simulation was initialised from the ECMWF operational analysis on the 1st of November 2018 and then ran freely until the sampled dates, detailed below for each case individually.

### Brightness temperature simulations

We compute the simulated brightness temperature perturbations from this dataset in a linearised fashion to save computation time. To that end, we must first remove the temperature background from the model temperature structures^27^. We then configure and run new 2D simulations using JURASSIC2. Unlike the regular spatial grid used for the observational filter calculation, we carry out these simulations using a coarser horizontal grid of spacing 0.025° in latitude and a vertical grid of 0.5 km, approximating the grid spacing used in the spectral IFS run.

We then compute the 2D Jacobian matrix (also called a sensitivity kernel) of a single brightness temperature measurement for a climatological atmosphere, to effectively linearise the complex forward model. By convolving this sensitivity matrix with 2D IFS-derived temperature perturbations individually along each 0.025°-spaced meridian, we directly compute the (linearised) brightness temperature perturbations which would be observed by an instrument at the height of the ISS travelling northward along each meridian. We repeat this for a range of sub-limb observing angles, across the range 25–90°.

### Gravity wave analysis

**Fig. 3:**
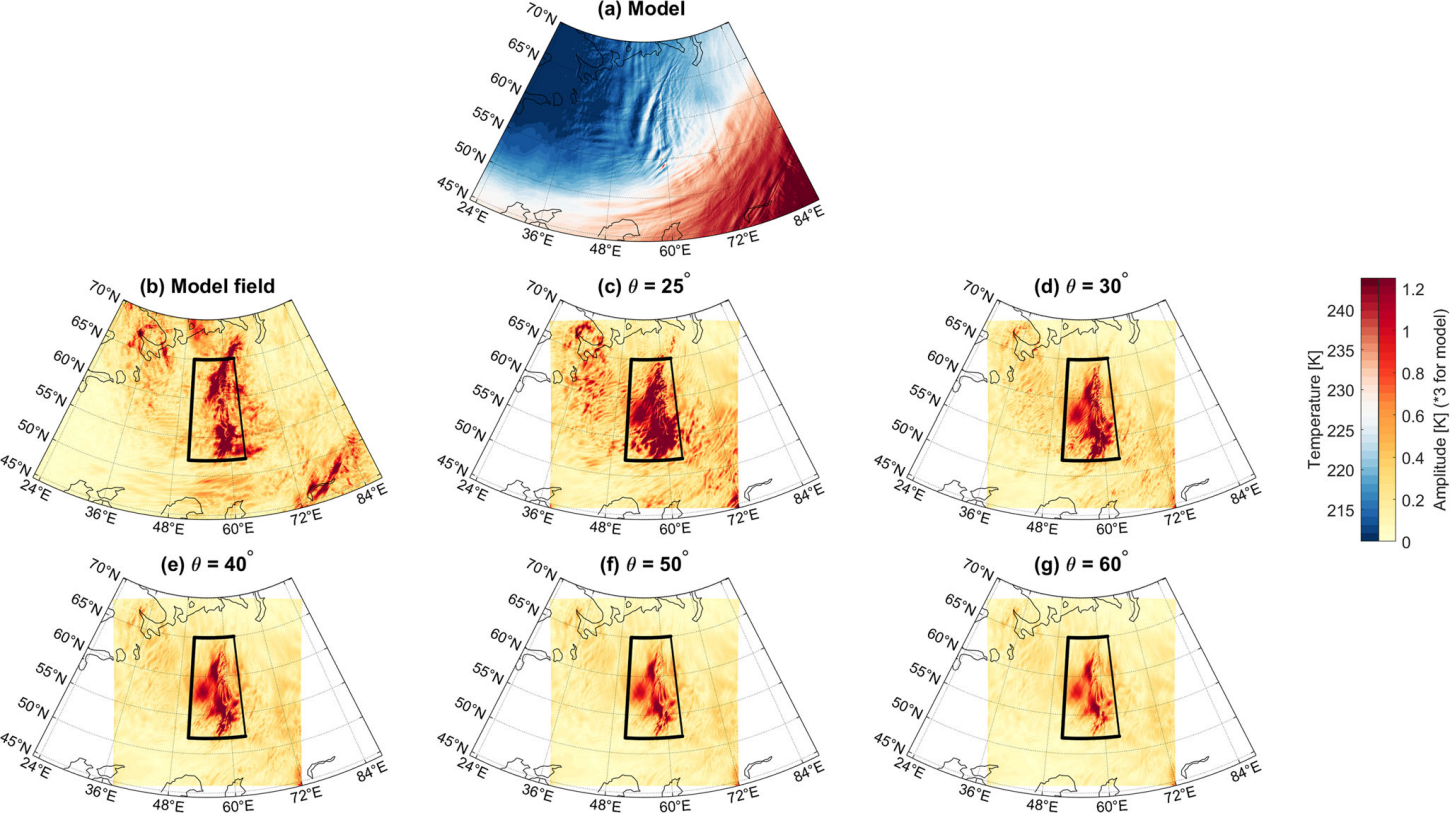
Temperature amplitudes recovered for a sample orographic wave over the Urals. **a** Temperature output at 37 km altitude of the TCo7999 simulations. **b**–**g** Estimated amplitude of this wave as measured using a 2D Stockwell Transform method, for brightness temperatures computed from model output sampled across a range of viewing angles. Black boxes show region averaged over to generate Fig. 5. Note that amplitude values in panel (**b**) have been downscaled by a factor of 3 to aid visual comparison (see text for details).

**Fig. 4:**
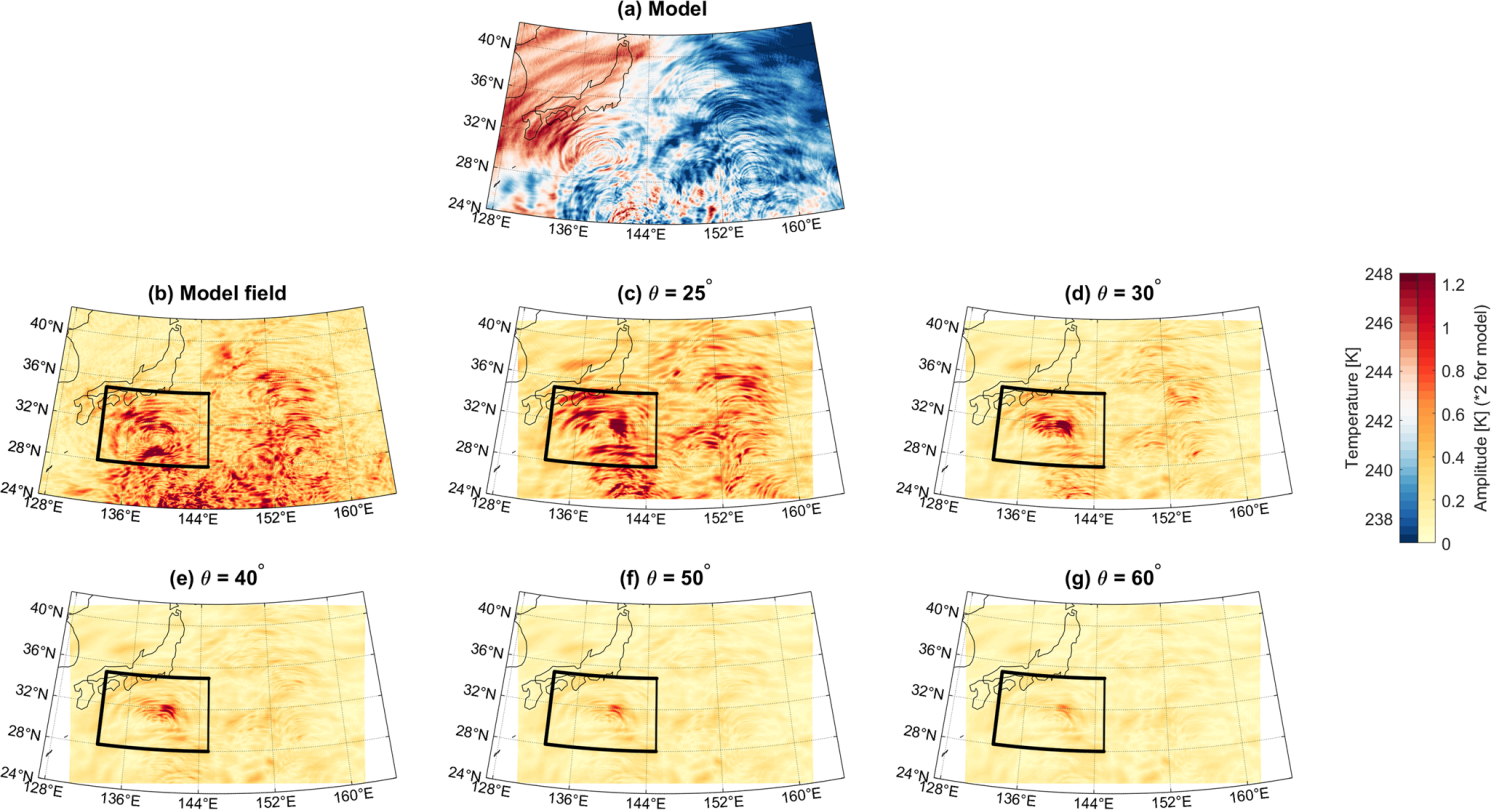
Temperature amplitudes recovered for a group of GWs associated with convection southeast of Japan. **a** Temperature output at 37 km altitude of the TCo7999 simulations. **b**–**g** Estimated amplitude of this wave as measured using a 2D Stockwell Transform method, for brightness temperatures computed from model output sampled across a range of viewing angles. Black boxes show region averaged over to generate Fig. 5. Note that amplitude values in panel (**b**) have been downscaled by a factor of 2 to aid visual comparison (see text for details).

The above step provides global estimates of brightness temperatures for a range of sub-limb observing angles and a backward-looking observer flying along the meridians. We now proceed to analyse these data for GW signatures, to assess the effect of varying the sub-limb angle on how well the GWs are recovered at each angle. The simulated observations are analysed using the two-dimensional Stockwell Transform in ref. ^28^, as modified for computational efficiency in refs. ^29,30^. Due to the very large volume of the data when considered at global scales, the computational complexity of applying techniques of this nature to such large volumes of data, and the brief format of this study we focus here on two cases: a large orographic wave over the Ural mountains (Fig. 3a) and a group of convective waves southeast of Japan (Fig. 4b). Other sampled cases provide results broadly consistent with these two examples.

We first interpolate the brightness temperature data onto a regular 1 × 1 km spatial grid centred around the GW feature of interest. Interpolation to a regular spatial grid is required as the S-Transform analysis is based on Fast Fourier Transform algorithms. For our Urals case, this grid is centred at 55°N, 115°E and extends ±25° in longitude and ±14° in latitude at mean longitude and latitude respectively, while for the Japan case, it is centred at 33.5°N, 146°E and extends ±12° in both longitude and latitude. We carry out this interpolation separately for data extracted at all viewing angles from 25 to 60°, and the results from each viewing angle assessed are shown individually.

We then apply the 2D ST to these data. This analysis is carried out on the regular spatial grid, which at the centre location has Cartesian *x* and *y* axes corresponding to the zonal and meridional directions. In both these directions, we use a scaling parameter *c* of 0.25^28,31^ and limit the maximum horizontal wavelength to 500 km, and we permit the analysis to find the strongest 1000 frequencies present in the data. These choices are consistent with previous studies using the ST. Figs. 3b–f and 4b–f show maps of the estimated wave amplitudes calculated using this approach.

### Case study results

We consider first the GW case over the Urals, Fig. 3. The phase fronts of this wave are aligned north-south, consistent with the north-south topography of the underlying mountain range, and this combined with the morphology and large amplitude of the wave gives significant confidence that this wave is orographic in origin. We see peak amplitudes >1 K across all viewing angles, falling from a peak amplitude of >3 K (note saturated colour scale) at the lowest viewing angle to ~1.25 K at 60°. As we vary the viewing angle of the instrument, the geographic location of the wave does not shift, and the morphology of the inferred amplitude field remains largely constant. The results seen for the sub-limb viewing cases, particularly at low viewing angles, are morphologically consistent with those obtained from an equivalent S-Transform analysis applied to the raw model data, but lower in magnitude. The largest sensitivity at large viewing angles is seen at 60°E, 54°N, where 3D analysis (not shown) indicates particularly low horizontal and long vertical wavelengths.

This case contrasts in several important ways with our Japanese case, Fig. 4. In this case, the observed phase fronts are radial in morphology, and are geolocated over similar features in the troposphere (not shown). They are therefore consistent with convective GW generation. While again the waves remain at the same location as the observation angle is increased, here the reduction in amplitude with increasing angle is much larger, falling from a peak amplitude ~1.25 K at 25° to <0.2 K at 60°. The exception, in this case, is a spot around 32°N, 140°E. There 3D analysis reveals northward propagating waves (phase fronts tilted to the instrument), with <50 km horizontal wavelengths and different vertical wavelengths up to 20 km, i.e., the steepest waves are visible for the largest angles.

**Fig. 5:**
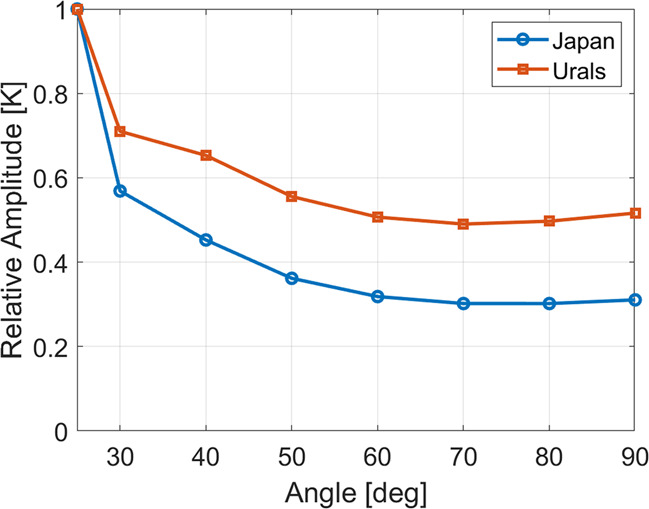
Reduction in recovered amplitudes relative to 25° case. Figure shows average GW amplitudes over the regions defined by black boxes in Figs. 3 (orange line) and 4 (blue line), as computed from brightness temperature estimates over a range of viewing angles.

Figure 5 shows the different amplitude reductions with sub-limb viewing angle in these two cases as comparative line plots, averaged over the black-outlined regions in Figs. 3 and 4. In both cases, the measured amplitudes have been normalised to the values calculated for the 25° sub-limb viewing angle (themselves ~50% reduced from the original model fields), in order to focus on the relative amplitude reduction as a function of angle. This highlights the rapid decline in signal strength for the convective case in comparison to the orographic case. While the amplitudes measured for the orographic case drop never below 50% of the best-case geometry considered, the convective case rapidly decreases, stabilising at around 30% of the original amplitude for all angles above ~50°.

## Discussion

Convective GWs have expected vertical wavelengths of 6–15 km and cover a huge range of horizontal scales. While nadir sounding instruments are almost insensitive to this wavelength range, limb sounders can resolve convective GWs excited by MCCs. Here the challenge is to accurately quantify momentum flux by bringing an IR limb imager into space^32,33^. Convective GWs from single towers, however, are not covered with the existing observation techniques.

Our results provide a clear example of the benefits of a sub-limb viewing geometry over a nadir or near-nadir geometry for observing the GWs produced by convective activity. In both the theoretical and realistic cases considered, the amplitudes of GWs with the spectral properties typical of convective wave activity are suppressed significantly at high sub-limb angles (i.e., near-nadir geometries) and much better recovered (although still reduced) at low sub-limb angles (i.e., sub-limb geometries).

Due to advances in detector technology since the work described in ref. ^24^, highly sensitive infrared detector arrays for wavelengths 4 μm with resolutions of order 1000 × 1000 pixels and frame rates ~100 frames/s are now available commercially. Since GW observations of the type needed for atmospheric science simultaneously require a wide angle range and a small field-of-view (FOV) for each pixel, a low orbit such as that provided by the International Space Station (ISS) is highly favourable. From this orbit, the distance from the observing platform to the saturation altitude of the measurement is between 450 and 1100 km depending on the viewing angle, and an FOV width perpendicular to the instrument line of sight (LOS) 0.3–0.6 km (neglecting diffraction) would cover a swath of width 300–600 km for downward viewing angles of 25–60°, respectively (Fig. 1, where *σ* is defined as the ‘sub-limb angle’ hereafter). Despite the high orbital velocity of the ISS platform, the image recording rates needed to prevent smearing from measurements with limited integration times are also easily achievable with current-day technology. We note however that sensitivity estimates depend on the speed of the optics and are thus beyond the scope of this brief study.

While the heavily reduced amplitude seen at the nadir is not a fundamental problem for wave detection in the idealised and simulated cases considered here, it is a major problem for real-world observations. Our idealised observational filter calculations are inherently noise-free, and our model-based analyses lack both retrieval and instrumental noise. In the real world, however, the presence of a noise floor means that GWs with suppressed amplitude fall below this floor. As such, these waves become functionally undetectable even if, theoretically, a nadir observing geometry should allow some signal to be recovered from them. This has hampered studying of this important source of atmospheric GWs, leaving the partitioning of GW momentum flux between GWs from MCCs and single convective cells one of the large controversies in atmospheric physics. As such, a sub-limb geometry like the concept proposed here has significant design benefits for the important scientific problem of characterising convective atmospheric gravity waves from single convective cells.

## Methods

To simulate measurements of the envisioned instrument, we employ the Jülich Rapid Spectral Simulation Code Version 2 (JURASSIC2) forward model^34^. This forward model uses the emissivity growth approximation^35^ to compute emissions over the spectral band from 2320 to 2340 cm^−1^. JURASSIC2 is capable of 2D and 3D simulations and inversions, and here we employ a simplified 2D forward model along the meridians, in order to reduce the computational complexity of the problem. As we wish to focus primarily on the effects of different viewing angles in the vertical, to further simplify the analysis we do not account for FOV effects, i.e., each brightness temperature is computed using an infinitesimally thin ‘pencil’ beam.

### Reporting summary

Further information on research design is available in the Nature Research Reporting Summary linked to this article.

## Supplementary information

Reporting Summary

## Data Availability

The observational filter simulations presented in Fig. 2 have been generated computationally as described in the text and associated references and do not incorporate any underlying data. As the underlying simulation output used in Figs. 3–5 is extremely large (42GB per 1-h timestep) we cannot easily archive these data separately for this study, but the retrieved brightness temperatures for the regions and times used in our study have been archived at Zenodo (10.5281/zenodo.6637647) as Matlab-format data files.
